# Atypical autoimmune hepatitis presenting as acute hepatitis with prominent cholestatic features: a case report

**DOI:** 10.3389/fimmu.2026.1787888

**Published:** 2026-05-21

**Authors:** Xiaomiao Yang, Jingdong Cui, Zhiyuan Xu, Xiaojuan Zheng, Yanfei Zhan, Tingting Lv, Xinyan Zhao, Junjie Ren

**Affiliations:** 1 Department of Gastroenterology and Hepatology, The First Hospital of Shanxi Medical University, Taiyuan, Shanxi, China; 2 Department of Liver Research Center, Beijing Friendship Hospital, Capital Medical University, National Clinical Research Center for Digestive Diseases, Beijing, China; 3 Shanxi Key Laboratory of Digestive Diseases and Organ Transplantation, The First Hospital of Shanxi Medical University, Taiyuan, Shanxi, China

**Keywords:** autoimmune hepatitis, drug-induced liver injury, acute cholestatic hepatitis, immunoglobulin G, glucocorticoid, azathioprine

## Abstract

Autoimmune hepatitis (AIH) poses distinct diagnostic challenges due to its heterogeneous clinical presentation and lack of disease-specific features. This is further complicated by drug-induced liver injury (DILI), which can present with laboratory and histological findings indistinguishable from those of AIH. Here, we report the case of an elderly man who presented with elevated liver enzymes of unclear etiology. Initial laboratory tests showed negative antinuclear antibody (ANA) and normal serum immunoglobulin G (IgG) levels. Based on his history of consuming herbal supplements and a liver biopsy showing acute cholestatic hepatitis with cholangitis lenta, a diagnosis of DILI was initially considered. During a 1-year follow-up, two recurrent episodes of elevated liver enzymes and urine discoloration were observed. Concurrent laboratory tests revealed seroconversion to ANA positivity and an IgG level exceeding 1.2 times the upper limit of normal (ULN). A follow-up liver biopsy demonstrated typical histopathological features of AIH. In summary, repeated evaluations—including two liver biopsies and a year-long clinical follow-up—confirmed the diagnosis of AIH. This case underscores the importance of considering AIH in patients who initially present with acute hepatitis with prominent cholestatic features, even when the early clinical picture is atypical.

## Introduction

Classical autoimmune hepatitis (AIH) is a chronic inflammatory liver disease that, if untreated, often leads to cirrhosis, liver failure, and death (1). It is characterized by female predominance, hypergammaglobulinemia, circulating autoantibodies, and a good response to immunosuppressive therapy. Incidentally, an acute atypical AIH diagnosis may be difficult since it exhibits negative serological manifestations and diverse pathological features. Sometimes, drug-induced liver injury (DILI) has features similar to those of other liver diseases, including AIH (2). DILI is rare in the general population but has become more prevalent in hospitalized patients, especially among patients with unexplained liver dysfunctions. The incidence of herbal and dietary supplements induced hepatotoxicity is increasing globally (3). Both diagnoses are reliant on overlapping clinical features. It is noteworthy that patients with drug-induced autoimmune-like hepatitis (DI-ALH) present similarly to AIH clinically, immunologically, and histologically (4). The only feature that may distinguish DI-ALH from AIH is the lack of relapse after discontinuation of the immunosuppression in DI-ALH. Here, we report a man with acute atypical AIH as a manifestation of acute hepatitis with prominent cholestatic features.

## Case presentation

The patient, a 58-year-old man, presented to the hospital on November 10, 2022, with jaundice and dark urine for 20 days. There was no history of fever, itchy skin, joint pain, rash, or other discomfort. His underlying diseases were hypertension, treated with oral amlodipine besylate tablets 2.5 mg once per day, and a history of mild fatty liver for 9 days. Personal history was negative, except for an occasional alcohol drinking history on a social basis only (<20 g/day). He acknowledged consuming dandelion (*Taraxacum mongolicum*) and apricot kernels (1–2 times/year) seasonally as herbal supplements. He denied other prescription drugs and over-the-counter drugs.

On physical examination, his BMI:body mass index was 25.9 kg/m^2^, and he had normal vital signs. His abdomen was soft and non-distended, with no hepatosplenomegaly.

Complete blood count (CBC), comprehensive metabolic panel (CMP), and coagulation panel (CP) were performed (**Table 1**). Serological testing indicated a resolved past hepatitis B virus (HBV) infection. HBV-DNA was undetectable. Infection evaluation, including viral hepatitis A, hepatitis C, hepatitis E, cytomegalovirus (CMV), Epstein–Barr virus (EBV), herpes simplex virus (HSV), and human immunodeficiency virus (HIV), was negative. Serum immunoglobulin G (IgG) was 15.22  g/L (8.6–17.4 g/L). Antinuclear antibody (ANA) and smooth muscle antibody (SMA) were negative by indirect immunofluorescence testing (IFT). Similarly, serum anti-mitochondrial antibody (AMA) and anti-mitochondrial antibody M2 subtype (AMA-M2), anti-liver kidney microsomal type 1 antibody (anti-LKM-1), anti-speckled protein 100 antibody (anti-SP100), anti-glycoprotein 210 antibody (anti-gp210), anti-liver cytosol type 1 (anti-LC-1), and anti-soluble liver antigen/liver-pancreas antigen antibody (anti-SLA/LP) were negative by Western blotting (WB). A computed tomography (CT) scan of the abdomen/pelvis demonstrated a periportal “tracking” sign and cholecystitis without cholelithiasis. Magnetic resonance cholangiopancreatography (MRCP) did not detect any evidence of biliary obstruction. Liver stiffness measurement (LSM) was 13.9 kPa with transient elastography (TE).

**Table 1:** Evolution of laboratory findings during long-term follow-up: CBC, CMP, and CP.

Laboratory studies	Normal range	Unit	Initial presentation	2 mo FU	5 mo FU	7 mo FU	12 mo FU	14 mo FU	36 mo FU
WBC	3.5–9.5	×10^9^/L	10.2	14.9	14.2	NA	19.4	13.6	9.4
RBC	4.3–5.8	×10^12^/L	3.99	4.42	5.13	NA	4.9	4.55	4.5
PLT	125–350	×10^9^/L	302	325	230	NA	319	158	246
ALT	9–50	U/L	363	47	1182	30	1417	24	13
AST	15–40	U/L	167	23	365	22	510	26	18
ALP	45–125	U/L	119	54	80	61	85	75	59
GGT	10–60	U/L	140	52	228	30	169	88	25
R-values	–	–	7.63	NA	NA	NA	NA	NA	NA
TBIL	0–23	μmol/L	214.3	20.7	33.1	16.6	48.6	19.5	13.4
DBIL	0–7.2	μmol/L	116.8	9.9	9.7	3.4	22.1	5.2	2.0
TP	65–85	g/L	56.2	NA	66.3	NA	69.3	66.7	73.5
ALB	40–55	g/L	31.0	NA	33.7	NA	35.8	37.6	48.9
PT	10–14	s	15.1	NA	14	NA	14.5	NA	NA
PT%	70–130	%	60.0	NA	67.8	NA	64	NA	NA
INR	0.8–1.2	–	1.3	NA	1.2	NA	1.25	NA	NA
Ferritin	30.06–400.2	ng/ml	1195.9	NA	NA	NA	734.7	NA	NA
SI	10.6–36.7	μmol/L	39.7	NA	NA	NA	43.1	NA	NA
CER	0.2–0.6	g/L	NA	NA	NA	NA	0.22	NA	NA
AFP	<10	ng/ml	47.05	NA	8.38	NA	31.23	NA	3.38
IgG	8.6–17.4	g/L	15.22	18.27	18	17.35	21.32	11.81	10.88
ANA	–	–	Negative	NA	Negative	Negative	1:40	NA	NA

WBC, white blood cell; RBC, red blood cell; PLT, platelet; ALT, alanine aminotransferase; AST, aspartate aminotransferase; ALP, alkaline phosphatase; GGT, gamma-glutamyl transferase; TBIL, total bilirubin; DBIL, direct bilirubin; TP, total protein; ALB, albumin; PT, prothrombin time; PT%, prothrombin time percentage; INR, International Normalized Ratio; SI, serum iron; CER, ceruloplasmin; AFP, alpha fetoprotein; NA, not available; FU, follow-up; ANA, antinuclear antibody.

**Figure 1:**
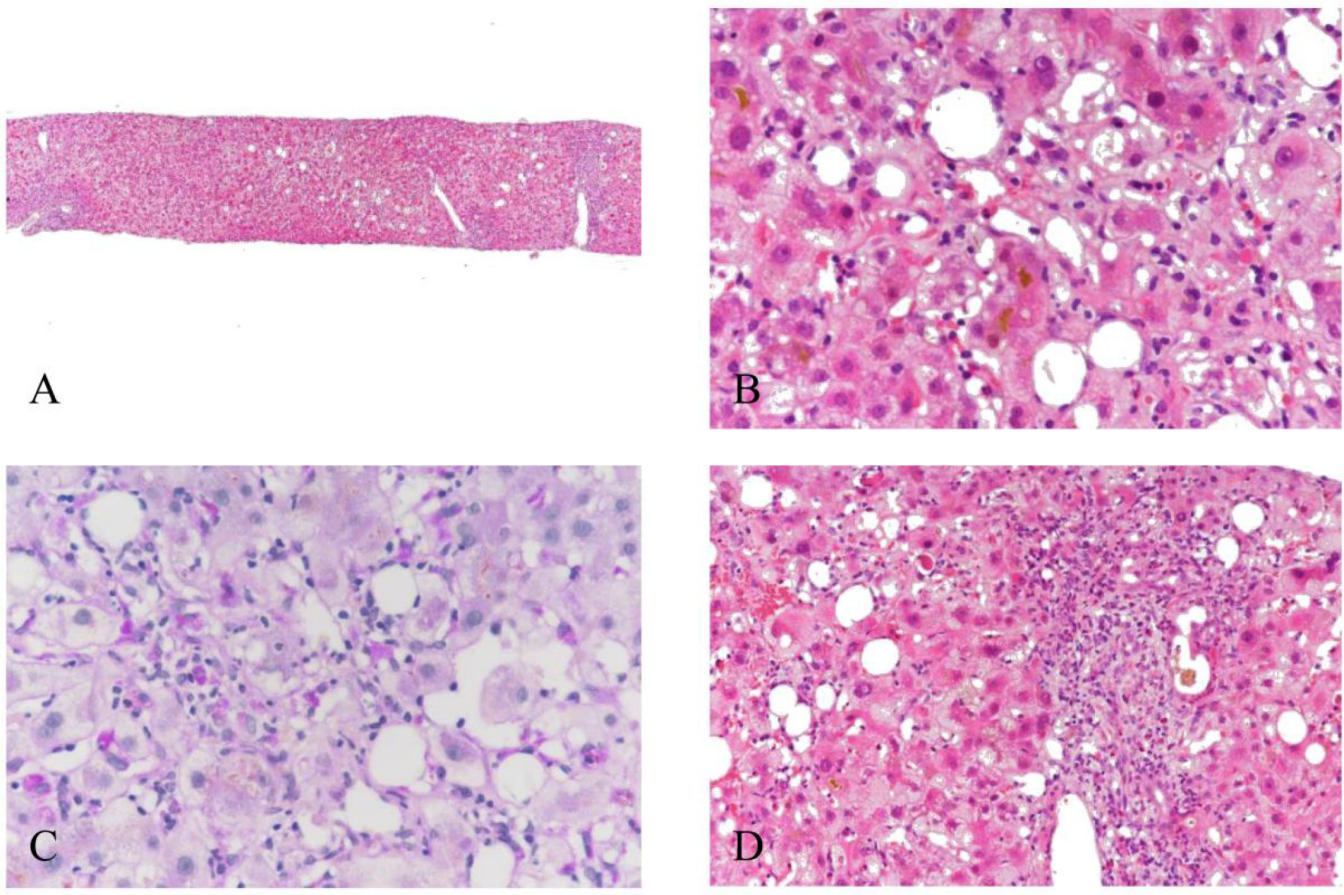
Liver biopsy showed acute cholestatic hepatitis with cholangitis lenta and mild fatty liver. Mild lobular architectural disarray was noted [**(A)**; H&E, ×40]. The dominant lesion was centrilobular (zone III) hepatocellular necrosis accompanied by cholestasis [**(B)**; H&E, ×400], including both hepatocellular cholestasis and bile canalicular cholestasis. Kupffer cells were hyperplastic and activated [**(C)**; DPAS, ×400]. Focal or spotty necrosis was seen within the lobules, and approximately 5%–10% of hepatocytes showed macrovesicular steatosis. Perisinusoidal fibrosis was distinctly prominent. Most portal tracts contained a mild mixed inflammatory infiltration with mild interface hepatitis. Small bile ducts were identifiable with inflammatory cell infiltration into the biliary epithelium. A mild-to-moderate ductular reaction was present, with occasional ductular ectasia containing bile plugs [**(D)**; H&E, ×200]. Portal tracts showed mild fibrous expansion. Copper and iron stains were negative. H&E, hematoxylin and eosin DPAS:periodic acid-schiff with diastase.

The patient underwent a liver biopsy. The biopsy was interpreted and confirmed by a specialist who is a pathologist and hepatologist. Liver biopsy showed centrilobular (zone III) hepatocellular necrosis accompanied by cholestasis and cholangitis lenta (5, 6). Focal or spotty necrosis and perisinusoidal fibrosis were seen within the lobules. Hematoxylin and eosin (H&E) stain highlighted mild interface hepatitis and mild-to-moderate ductular reaction. Overall, the liver histology revealed acute cholestatic hepatitis and mild fatty liver (**Figure 1**).

A Roussel Uclaf Causality Assessment Method (RUCAM) score of 4 indicated “possible” causality for herb-induced liver injury (HILI). Based on the symptoms, signs, and examination, the clinicians considered that the primary diagnosis was HILI (hepatocellular, acute) (7). Then, the patient was advised to discontinue suspected dandelion (*T. mongolicum*) and apricot kernels. He received symptomatic treatments including choleretic (ursodeoxycholic acid) and hepatoprotective agents (glutathione and magnesium isoglycyrrhizinate). The patient’s symptoms and liver function indicators improved (**Table 1**).

## Outcome and follow-up

During the follow-up period, the patient strictly adhered to a low-fat diet and avoided all potential liver-damaging factors. However, at the 5-month follow-up, the patient was readmitted for dark urine; liver function tests revealed re-elevated transaminase levels (**Table 1**). Other laboratory indicators and CT scan showed no significant changes compared to previous results. Liver function normalized within 2 months with the same initial treatment (**Table 1**).

**Figure 2:**
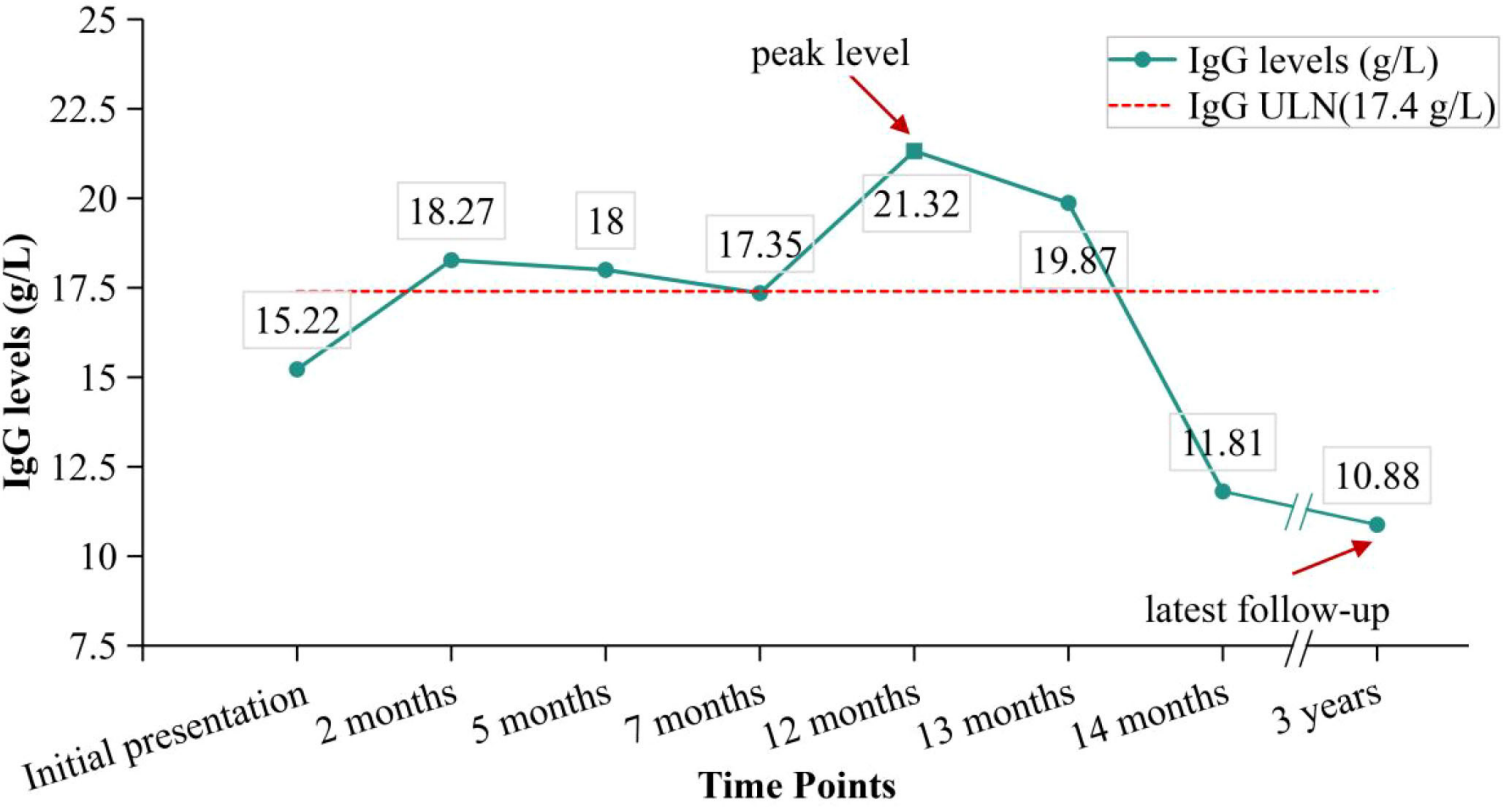
Changes in IgG levels. ULN, upper limit of normal.

**Figure 3:**
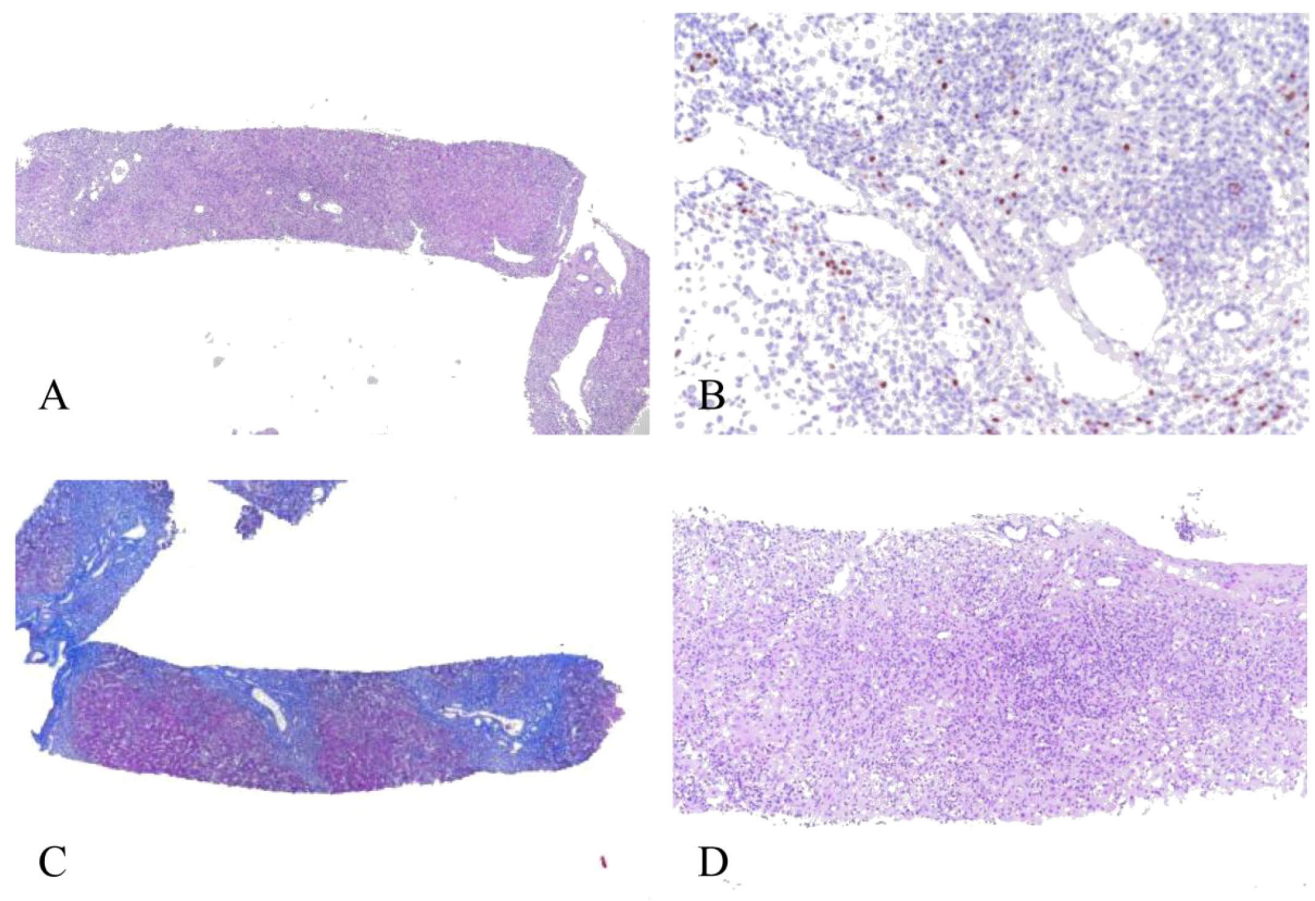
The second liver biopsy showed AIH (G4 S2–3) with disrupted lobular structure [**(A)**; H&E, ×40]. The main lesions were the expansion of most portal areas, moderate-to-severe inflammatory cell infiltration, mainly mononuclear cells, moderate-to-severe interface hepatitis, and readily identifiable plasma cells [**(B)**; MUM-1, × 200]. Portal-to-portal and portal-to-central bridging necroses were evident; small bile ducts were discernible but exhibited irregular epithelial arrangement; moderate ductular reaction can be observed, and some small bile ducts differentiated into hepatocytes to form rosettes. Fibrous tissue proliferation in portal tracts formed a bridging fibrous septum, separating hepatocytes and resulting in lobular structural disorder [**(C)**; Masson, ×40]. Multilobular necrotic collapse was present, with zone III centrilobular hepatocyte necrosis and confluent necrosis. Focal lobular necrosis was evident, accompanied by prominent inflammatory cell infiltration and sinusoidal macrophage activation [**(D)**; H&E, ×100]. Immunohistochemical staining for IgG4 was negative. H&E, hematoxylin and eosin; AIH, autoimmune hepatitis.

The patient remained stable at regular outpatient follow-ups. His ANA remained consistently negative, while IgG showed intermittent elevation (**Figure 2**). However, he was re-hospitalized for severe liver dysfunction at the 1-year follow-up (**Table 1**). He had dark urine on physical examination. Abdominal examination was negative. Elevated IgG levels (21.32 g/L) with elevated IgA levels (4.47 g/L) and positive ANA (1/40) were detected, raising the suspicion of AIH. LSM was 34.5 kPa with TE, which may indicate inflammation. CT and MRCP findings were essentially unchanged compared with prior imaging. He underwent a second liver biopsy, which showed AIH (G4 S2–3) with extensive mixed inflammatory infiltration, moderate-to-severe interface hepatitis, and few hepatocyte rosettes (**Figure 3**).

His score for AIH according to the 2008 “simplified” histological criteria of the International Autoimmune Hepatitis Group (IAIHG) was above 6 (probable diagnosis for a score of 6 and definitive diagnosis for scores >6) (8). The patient was started on methylprednisolone 40 mg once daily (qd) with slow tapering of 4 mg per week. Azathioprine 50 mg was added after 2 weeks of treatment. His liver serology improved after 2 months (**Table 1**). He is currently stable with normal transaminases on treatment with methylprednisolone 2 mg and azathioprine 50 mg qd (**Table 1**). At the 3-year follow-up, LSM decreased markedly to 11.4 kPa from the second relapse.

## Discussion

AIH is an immune-mediated liver disease of unknown etiology that can occur in patients of any age, sex, or ethnicity (8). The patient’s history, the evolution of laboratory findings, and the liver biopsy results collectively suggest that acute atypical AIH was the most probable diagnosis. The typical presentation of AIH tends to be chronic with mild elevation of AST and ALT levels; however, it is essential to recognize acute atypical AIH because of its atypical presentations. According to EASL:European Association for the Study of the Liver guidelines, TE is unreliable during acute hepatitis. The value of LSM as a predictor of outcome in patients with AIH remains to be determined (9). Histological examination of the liver is an important requisite for early diagnosis of acute-onset AIH, even though patients do not always show typical features. In view of the strong suspicion of autoimmune etiologies and HILI, the patient underwent a liver biopsy, which was interpreted as acute cholestatic hepatitis and mild fatty liver. The lack of typical AIH clinicopathological features in this case hampers early and accurate diagnosis. Fortunately, the second liver biopsy histology revealed the typical pathological manifestations of AIH. He was started on standard treatment strategies and received biochemical remission. We emphasize the need for liver biopsy once the common etiologies are ruled out, regardless of the presence of AIH-specific antibodies and the serum IgG level.

Our case was initially diagnosed with HILI because of its atypical clinicopathological manifestations. DILI has a relatively low incidence, whereas the incidence of HILI remains under investigation. Currently, there is no conclusive evidence that dandelion (*T. mongolicum*) and apricot kernels can cause liver injury. Hepatocellular-type DILI and acute AIH exhibit substantial clinical overlap, making them difficult to distinguish (10). Moreover, most patients with hepatocellular injury are taking one or more drugs and herbal or dietary supplements that could potentially damage the liver, and autoantibodies, including ANA, are sometimes detected in patients with DILI (11). First, long-term follow-up observations for recurrence can assist clinicians in reaching a diagnosis when faced with the difficulty of distinguishing between DILI, AIH, and DI-ALH. Second, the majority of DILI patients demonstrate normalization of liver function following discontinuation of the suspected drugs (12). Last, according to the EASL guidelines, for patients with initial onsets, a clear medication history, and marked autoimmune characteristics that cannot be diagnosed, even after the discontinuation of the suspected drugs, immunosuppressive therapy can be considered and gradually tapered following clinical improvement. If no recurrence is observed during follow-up, a diagnosis of DILI or DI-ALH can be confirmed, whereas disease recurrence without drug re-exposure supports a diagnosis of AIH (7).

Acute presentation of AIH occurs in 22%–43% of all AIH cases, and is not a rare condition. Of note, the prevalence of IgG elevation is lower in patients with an acute presentation and ranges from 25% to 39% (13, 14). Similarly, ANA/SMA positivity is often absent in the acute presentation of AIH patients. On the other hand, previous evidence suggests that in acute AIH, clinical and histological findings typically described in chronic AIH could be less frequent (13). The EASL guidelines recommend first-line screening by IFT on triple rodent tissue (kidney, liver, and stomach) for ANA, SMA, anti-LKM-1, and anti-LC-1, in parallel with anti-SLA/LP by solid-phase assay. If IFT is negative, retesting at a lower dilution (1:40 in adults) is advised; ELISA on HEp-2 cells is an accepted alternative with validated cut-offs (8). In this patient, ANA was negative on HEp-2 cell IFT, while SMA was negative by tissue-based IFT (rat kidney and stomach). Serum anti-LKM-1, anti-LC-1, and anti-SLA/LP were negative by WB. Such variations in detection methodologies may account for the potential omission of borderline positive autoantibody results. It is imperative that clinicians engage closely with immunology laboratories to develop and implement unified, standardized testing protocols for autoantibody detection.

There are no clear-cut algorithms or clear diagnostic histological criteria for acute AIH (15). A new 2022 consensus recommendation for histological criteria of AIH was established by the International AIH Pathology Group (IAIH-PG) ,which first considered the acute presentation of AIH (16). The main highlight of this consensus scoring was the inclusion of acute AIH cases as lobular hepatitis with or without centrilobular necroinflammation, with hepatocyte necrosis being recognized as part of the histological spectrum of AIH, which, till then, had been conventionally associated with DILI (17). Lobular inflammation was evident at the first liver biopsy in this patient, accompanied by mild interface hepatitis and cholangitis lenta. His score for AIH according to the 2008 IAIHG simplified histological criteria was 2, making AIH less likely in the initial differential. However, according to the 2022 IAIHG-PG consensus recommendations, the patient fulfilled the criteria for likely AIH. Furthermore, a retrospective analysis demonstrated that the 2022 consensus recommendation may be more sensitive in the diagnosis of AIH compared with the 2008 “simplified” histological criteria (p < 0.001), consistent with our case (18).

We hope that this case improves the index of suspicion among hepatopathologists and clinicians for acute atypical AIH, especially presenting as acute hepatitis with prominent cholestatic features.

## Conclusions

This case report suggests that the serological and histological features of acute atypical AIH may vary during disease progression. This case also illustrates the complexity of differentiating acute atypical AIH from other entities such as classic AIH, DI-ALH, and other forms of DILI. Larger studies are needed to better define the rare pathological features of acute atypical AIH, including cholestatic hepatitis.

## Data Availability

The original contributions presented in the study are included in the article. Further inquiries can be directed to the corresponding author.
